# Crosstalk between PRLR and EGFR/HER2 Signaling Pathways in Breast Cancer

**DOI:** 10.3390/cancers13184685

**Published:** 2021-09-18

**Authors:** Raghuveer Kavarthapu, Rajakumar Anbazhagan, Maria L. Dufau

**Affiliations:** Section on Molecular Endocrinology, Division of Developmental Biology, Eunice Kennedy Shriver National Institute of Child Health and Human Development, National Institutes of Health, Bethesda, MD 20892, USA; kavarthapur@mail.nih.gov (R.K.); raj.anbazhagan@nih.gov (R.A.)

**Keywords:** breast cancer, PRLR, EGFR, HER2, signaling pathways

## Abstract

**Simple Summary:**

Prolactin receptor (PRLR) and epidermal growth factor receptor (EGFR/ERBB) signaling pathways have a crucial role in the development of mammary glands and breast carcinogenesis. In this review, we have provided ample evidence to support the significant role for PRLR and EGFR/HER2 signaling crosstalk in breast cancer. Recent studies from our lab and other finding also recognize how these two receptors work together to induce hyperactivation of their downstream signaling kinases that overlap with each other and in turn further induce target genes and other pro-oncogenic factors. Prolactin through PRLR can activate both EGFR and HER2 downstream signaling pathways which in turn leads to activation of other oncogenic growth factors that promote growth, survival, and proliferation of breast cancer cells. The crosstalk between PRLR and EGFR/HER2 signaling is an additional route that magnifies the actions of their ligands, and further promote tumor growth. This may be responsible for endocrine resistance in breast cancer patients resulting in tumor relapse.

**Abstract:**

Prolactin receptor (PRLR) and epidermal growth factor receptor (EGFR/ERBB) signaling pathways activated by prolactin (PRL) and epidermal growth factor (EGF), have a major role in the mammary gland development and in the etiology of breast cancer, respectively. ER+ breast tumors comprise up to 75% of all breast cancers and 10% of these are HER2+. Elevated levels of PRLR in breast tumors, high circulating levels of PRL and increased expression of ERBB1/2 in patients that become resistant to endocrine therapy have shown to be associated with higher risk of cancer progression. In this review, we examine the role of crosstalk between PRLR and ERBB1/2 signaling pathways in the activation of unliganded ERα, cyclin-D1 and other oncogenic factors (MYC, FOS, JUN) in breast cancer. PRL/PRLR and EGF/EGFR induces phosphorylation of ERα through activation of MEK/MAPK and PI3K/AKT signaling pathways. PRL in breast cancer cells via PRLR/JAK2 can also induce phosphorylation of ERBB2/HER2, which in turn activates the downstream RAS/MEK/ERK pathway required for ERα phosphorylation. EGFR, independent of PRL/PRLR, can activate STAT5 indirectly via c-SRC and drive the expression of target genes involved in cell proliferation and survival. The crosstalk between PRLR and HER2, where PRL induces HER2 signaling can be an alternative route for ERα activation to induce transcription of PRLR and other ER target genes. We believe that overexpression of EGFR/HER2 and PRLR in breast tumors could maximize the actions of their ligands, and further induce cell proliferation promoting malignancy. This could also explain the resistance to endocrine therapy resulting in tumor growth.

## 1. Introduction

**Figure 1:**
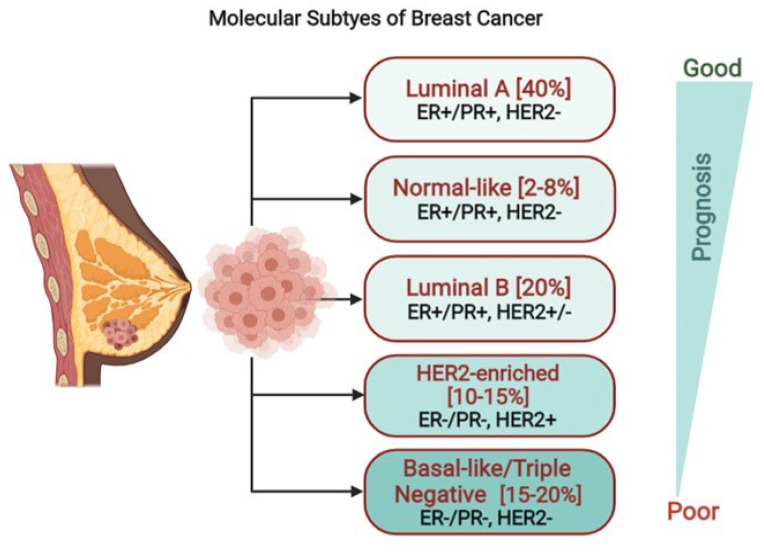
Molecular subtypes of breast cancer. Breast cancer can be categorized five different subtypes based on their expression of hormone receptors [estrogen receptor (ER) and progesterone receptor (PR)], and the HER2 overexpression. Prognosis varies based on the subtype of breast cancer where the triple negative cancer has worst prognosis and luminal A cancer has best prognosis. Targeted therapies, such as herceptin (aimed at the HER2 protein) and tamoxifen (aimed at the ER), can be used in the treatment of certain breast cancer subtypes. This image was created using Biorender software.

Epidermal growth factor receptors (EGFR/ERBB) and prolactin receptor (PRLR) signaling have an important role in mammary gland morphogenesis and carcinogenesis. Both these receptors are well known to induce and promote breast tumor via activation of their downstream signaling pathways. Breast cancer is the second leading cause of cancer-related death among women worldwide [1]. The majority (75%) of breast cancer patients are estrogen receptor (ER) positive. Based on expression/lack of expression as well as overexpression of certain receptors, breast cancer is classified into five subtypes which include luminal A, luminal B, HER2-enriched, triple negative/basal-like and normal-like [2] (Figure 1). The luminal A, B, and HER2+ breast cancer types are positive for ER or progesterone receptor. HER2 overexpression is only noticed in luminal B and HER2-enriched subtypes. The luminal A and B breast cancers are most prevalent subtypes (encompass 60–70% of all breast tumors) with good prognostic value derived from the luminal epithelium of breast ducts and are treated using anti-estrogen therapies (endocrine therapy) that target estrogen-mediated activation of the ER [2]. The triple negative breast cancer (TNBC) is the most aggressive subtype, poorly prognosed, more often observed in younger women and highly associated to BRAC1 mutations. The HER2+ breast cancer subtype that constitutes 15–20% of invasive breast cancers has an amplification/activation of the HER2 gene which results in overexpression of the HER2 receptor on the surface of breast cancer cells [2,3]. HER2 gene amplification or overexpression of HER2 receptor in breast tumors serves as prognostic biomarker and has been linked to poor patient survival [4]. HER2+ metastatic breast cancer patients receive trastuzumab, a monoclonal antibody, as a first line of treatment. Binding of trastuzumab to the external domain of HER2 receptor prevents HER2 homo- and heterodimerization and leads to disruption of downstream HER2 signaling pathways, blocking proliferation of the breast cancer cells [3,5,6,7]. Another hormone receptor which is overexpressed in most of the ER+ breast tumors is the PRLR. The role of PRLR in the etiology and proliferation of breast carcinoma induced by prolactin (PRL) has been well established. Prospective studies reveal up to 95% of female breast tumors, and 60% of male breast carcinomas express high levels of PRLR [8]. In large epidemiological studies it has been well documented that elevated circulating PRL levels are correlated with increased breast cancer risk and metastasis in premenopausal women and this risk is greater for ER+ breast cancer and lymph node-positive tumors [9,10]. PRL also facilitated risk prediction of invasive breast cancer in postmenopausal women [11]. Some clinical studies have highlighted that the expression of PRLR can be used as a favorable prognostic marker for benign breast tumors [12,13] and aggressive type of luminal breast cancer [14]. 

Contrary to the believed role of PRLR in breast cancer progression, recent studies have indicated that PRL exerts anti-tumorigenic effects in HER2 positive breast cancer cells through regulation of stemness [15,16]. Other recent study indicated that PRLR also acts as a driver of precise luminal and epithelial differentiation limiting cellular plasticity, stemness, and tumorigenesis. Loss of PRLR in HER2+ cancer cells enhanced tumorigenesis, metastasis, and resistance to therapy [17]. In another study, it has been shown that PRL circulating levels and PRLR signaling pathway aid as a sub-classifier and predictor of pro-differentiation therapy in TNBC [18].

The relevance of autocrine PRL in tumor initiation and its role in breast cancer progression has been well studied in transgenic mice models. NRL-PRL transgenic mice which mimic autocrine PRL effects develop aggressive mammary tumors of luminal B subtype [19]. Several mouse tumor models have somatic KRAS alterations associated with increased p-ERK1/2, decreased signal transducer and activator of transcription 5 (STAT5), and variable PRL levels [20,21,22]. Constitutive PRL signaling in mammary glands affects the tumor environment by altering the RAS pathway activation and induces aggressive tumors [22]. Interestingly, endogenous PRL induces ER responsiveness and enhances PRLR expression/transcription resulting in proliferation of breast cancer cells [23,24]. In addition, PRL can also induce non-classical and unliganded ER signaling. In MCF-7 cells, PRL and estrogen enhance AP-1 activity through phosphorylation of p38, ERK1/2, and c-Fos [25]. Conversely, estrogen can alter PRL-induced signaling by regulating the transcription of PRL in breast cancer cells and in rat pituitary cells [26,27]. Estrogen can also increase PRL-activated Stat5 activity in breast cancer cells [28]. More recently, we demonstrated the role of endogenous PRL in the upregulation of PRLR transcription induced by ERα in MCF-7 cells and requisite participation of STAT5a and complex formation with Sp1/C/EBPβ at the PRLR hPIII promoter [24]. PRLR is involved in the activation of MAPK/ERK signaling in several breast cancer cell lines, which mediate PRL-induced biological activity in these cells [29,30,31]. In addition to mammary carcinoma, PRLR signaling is also implicated in the development of prostate, colon, ovarian and endometrial cancers. PRL induces PRLR-mediated Jak2-STAT signaling in prostate cancer, and in colon cancer by modulating Notch signaling in a Jak2-STAT3/ERK manner [32,33]. Higher expression levels of PRLR were found in ovarian and endometrial tumors. PRL can also induce proliferation in endometrial and ovarian cancer cell lines [34]. In this review we highlight the crosstalk between downstream signaling cascades activated by PRLR and EGFR/HER2 in breast cancer and discuss how these receptors work together to affect breast cancer behavior and promote cancer progression. This review on the crosstalk between PRLR and EGFR/HER2 receptors activating several overlapping signaling cascades might explain their role in resistance to endocrine therapies often resulting in relapse, however further studies are warranted in clinical samples and using mice xenograft models. Also, provides a rationale for the use of combined therapeutic inhibitors targeting receptors and/or associated signaling pathways in the treatment of invasive breast cancer.

## 2. PRLR Signaling Pathway

**Figure 2:**
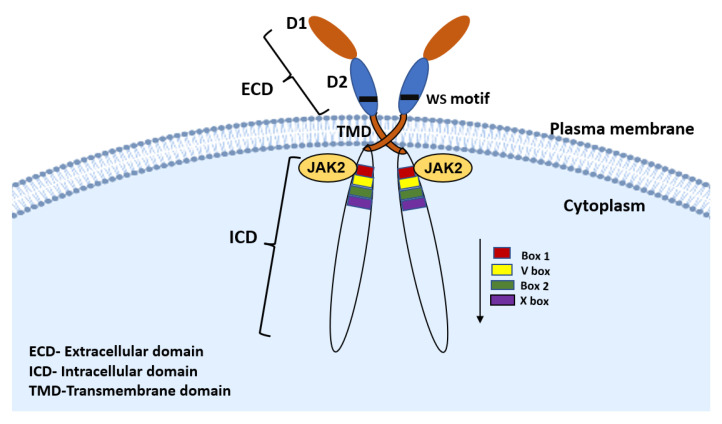
Structure of prolactin receptor. The PRLR consists of three domains, the extracellular domain, which contains a WS motif that acts as a molecular switch for activation, a transmembrane domain, and an intracellular domain showing Box 1 and Box 2 domains. Box 1 interacts with JAK2 and SRC family kinases.

PRLR belongs to the lactogen/cytokine receptor family which mediates several cellular actions of PRL in different target tissues. PRLR has three main domains, an extracellular, transmembrane and an intracellular domain. The extracellular domain is divided into two fibronectin domains, D1 and D2. The WS motif (Trp-Ser-Xaa-Trp-Ser sequence) in D2 acts as a molecular switch during ligand-bound activation of PRLR [35]. The intracellular domain of PRLR includes Box-1 and Box-2 domains (Figure 2). The Box-1 is known to interact with JAK2 and Src family kinases such as FYN. When PRL binds to PRLR, it causes receptor dimerization, resulting in the activation of classical JAK-STAT pathway and other signaling cascades including PI3K/AKT and RAF/MEK/ERK [35]. The phosphotyrosine residues of the PRLR act as docking sites for SHC/GRB2/SOS adapter proteins connecting the receptor to the RAS/RAF/MAPK signaling cascade [35]. PRLR signaling can also be mediated through the SRC family of kinases like c-SRC and focal adhesion kinases (FAK). PRL/PRLR induces phosphorylation of PI3K kinase via c-SRC, activating PI3K/PDK1/AKT/mTOR signaling which promotes translation of target proteins and cell survival [36,37]. PRL/PRLR signaling has an important role in mammary gland epithelial cell proliferation and milk production. PRL activates JAK2-STAT5 signaling pathway stimulating transcription of milk protein genes and the genes involved in cell proliferation like cyclin-D1 [36]. Studies from our lab and others in T47D and MCF-7 breast cancer cells have shown that PI3K/AKT and RAF/MEK/ERK pathways are activated in parallel following PRL treatment, which leads to profound cell proliferation and survival [30,31]. PRLR can also induce MAPK/ERK signaling cascade via PI3-kinase-dependent RAC/PAK/RAF/MEK pathway, which is in turn controlled by JAK2, SRC family kinases and FAK [37]. PRLR-mediated MCF-7 cell growth and migration was significantly abolished using inhibitors of RAC/PAK [37]. Several studies using breast cancer cells have shown that PRL activates unliganded ERα through phosphorylation at Ser118 and Ser167 residue. The activation of ERα promotes ligand-independent transcriptional initiation of ERE dependent target genes which seems to be an important factor in the proliferative and transcriptional actions of PRL in breast cancer cells [31,38,39]. In addition, we found that ERα upon activation by PRL/PRLR forms a complex with Sp1 and cEBPβ transcription factors. The participation of the ERα-c/EBP-Sp1 complex at the human PIII promoter region stimulates PRLR transcription/expression independently of estradiol [26,31]. In addition to the role of the predominant long form of PRLR in breast cancer, recently it has been shown that the human intermediate PRLR (alternatively spliced isoform) is a mammary proto-oncogene capable of stimulating cell survival and proliferation [40]. The oncogenic effect of intermediate PRLR isoform reaches to its full potential when expressed in concert with PRLR long form in MCF-10A cell lines. Heterodimers of long and intermediate PRLR isoforms displayed greater stability than PRLR long form homodimers. These can activate JAK2/RAS/MAPK pathway, but unable to induce STAT5A phosphorylation [40].

The most important transcription factor in PRLR signaling is STAT which regulates the growth, differentiation, and survival of mammary tissue. STATs are found to be activated/overexpressed in several types of cancers including breast cancer [41]. Constitutively activated STAT3 and STAT5, were shown to directly contribute to oncogenesis by stimulating cell proliferation and preventing apoptosis in various cancers. STAT5 could act as both a tumor suppressor and an oncogene in breast cancer under different circumstances [41]. The upregulation of STAT signaling promotes tumor growth and survival due to the inhibition of apoptosis, increased cell proliferation, migration, and dysregulated immune surveillance by promoting the expression of target genes like cyclin D, Bcl-2 and MMP-2 [42,43]. In ER-positive breast cancer, STAT5 expression enhanced the response to hormone therapy and increased the overall survival of patients [44]. In the established cancers, STAT5 get progressively inactivated with the progression to metastatic breast cancer due to enhanced regulation by tyrosine phosphatases [44,45].

PRL/PRLR signaling can also influence the breast tumor microenvironment through extracellular matrix components. PRLR signaling enhances breast cancer cell motility by regulating actin cytoskeleton rearrangement which involves phosphorylation of c-SRC, moesin, and FAK kinases by PRL [46]. PRL/PRLR can substantially promote migration and invasion of T47D breast cancer cells by activating alternate downstream effectors like TEC and NEK3 kinases, leading to cytoskeletal and focal adhesion rearrangements necessary for cell adhesion and migration [47]. Many breast tumors are characterized by reduced STAT5, high levels of PRLR expression, and MAPK signal components including AP-1 and pro-invasive matrix metalloproteinases [48]. Often breast tumors are highly invasive and are resistant to anti-estrogen treatment and chemotherapy. We believe the tumor micro-environment may be responsible for directing a particular PRLR signaling pathway of choice. The stiffness of collagen matrices has been identified as a key factor in PRL-PRLR-pathway choices [49]. In invasive breast cancer, there is a shift in PRLR signaling from STAT5-mediated pathways to FAK and MAPK pathways in a stiff collagen matrix and thereby favoring proliferation [49,50].

## 3. ERBB Signaling Pathway

ERBB family comprises four related tyrosine-kinase receptors, which include EGFR/ERBB1/HER1, ERBB2/HER2, ERBB3/HER3, and ERBB4/HER4. All members of the ERBB family share a common structure comprises of cysteine-rich extracellular domain, a transmembrane domain, and an intracellular tyrosine kinase domain with several phosphorylation sites. EGFR may initiate cellular signaling cascades by itself through homodimerzation or through transactivation with other ERBB family members via various ligands that can induce specific heterodimerization [51]. ERBB receptors are activated by several ligands, including EGF, TGF-alpha, amphiregulin for EGFR/ERBB1, and neuregulins for HER3 and HER4. Ligand binding causes receptor dimerization, activation of the kinase domains, auto-phosphorylation, and initiation of down-stream signaling pathways that regulate cell proliferation, survival, motility, invasion, adhesion, and angiogenesis. HER2 has no known ligand and its activation depends on heterodimerization with ligand activated ERBB members, or homodimerization when overexpressed in breast cancer [51,52]. HER3 having no kinase domain relies on another member of the ERBB family for target action. Hence, both HER2 and HER3 rely on the heterodimerization with other ERBB receptors to initiate specific cellular actions. The transphosphorylation of the ERBB dimer partners through activation of HER2 and EGFR stimulate various intracellular pathways including RAS/RAF/MEK, PI3K/AKT/mTOR, Src kinases, and STAT transcription factors [51]. Both EGFR and HER2 can activate STAT3 via phosphorylation through SRC promoting cell survival [53]. HER2 as a homodimer or a heterodimer with EGFR mediate key pathways downstream of it which include PI3K and MAPK signaling. The MAPK cascade includes RAS/RAF/MEK/ERK1/2 signaling kinases, which together activate specific transcription factors like c-MYC, c-JUN, and FOS resulting in cell proliferation. The PI3K/PDK/AKT/mTOR signaling phosphorylate downstream targets like p70 ribosomal protein S6 kinase 1 stimulates protein biosynthesis and can also promote cell proliferation mediated by inactivation of the p27/p21 cell cycle inhibitor [51]. In addition to cell proliferation, ERBB signaling modules can also mediate various other cellular activities, including angiogenesis, cell adhesion, cell motility and organogenesis [54,55]. EGF is a potent activator of ERK5 which is shown to promote cell proliferation of MCF-10A non-malignant breast epithelial cells. In contrast to ERK1/2, EGF-mediated activation of ERK5 occurs independently of RAS and requires MEK5 in breast cancer cells [56]. Interestingly, it has been shown that MEK5-ERK5 signaling promotes hormone-independent tumorigenesis of breast cells [57]. ERK5 is overexpressed in 20% of breast cancer patients and its activity is constitutively high in tumors overexpressing HER2 which can be a useful prognostic marker for disease-free survival [58]. Also, of note is that inhibition of ERK5 decreased cell proliferation and sensitized breast cancer cells to the action of anti-HER2 therapies [58].

## 4. PRLR and EGFR Signaling Crosstalk in Breast Cancer

Although PRLR and ERBB receptors are critical regulators of normal developmental processes, such as growth and morphogenesis of mammary glands, it has become increasingly evident that their dysregulation, because of overexpression, and/or mutations, leads to the development of cancer. Several studies have indicated that overexpression of PRLR, EGFR and HER2 receptors in breast tumors promotes tumor progression via their downstream signaling pathways [11,59,60,61,62]. In a recent study performed in patient breast tumor samples, co-expression of PRLR with TGFbR was observed in HER2 overexpressed (HER2+) and luminal breast cancer molecular subtype. Higher expression levels of PRLR were found in HER-2, luminal A and luminal B breast cancer subtypes [11]. In postmenopausal women with increased PRL and PRLR there is an increased risk of developing breast tumors and metastasis. PRLR is expressed at a higher level in tumors when compared to surrounding normal tissues with maximal expression in 70–95% of primary breast tumors [63,64,65]. Activation of PRLR promotes mammary tumor development in transgenic mice overexpressing PRL gene [59]. EGFR/ERBB1 overexpression is observed in 15–30% of breast cancer patients and is linked to tumor size and bad clinical outcomes [62] HER2 is also overexpressed in 20–30% of breast cancers but particularly in ductal carcinoma is attributed to its gene amplification, and results in constitutive activation of HER2 signaling, associated with poor clinical outcome and disease progression [52]. Moreover, EGFR and HER2 amplification after breast cancer surgery favor metastasis and it has a considerable prognostic value as biomarker for invasive breast carcinoma [66]. Amplification of HER2 facilitates hyperactivated signaling of RAS/RAF/MAPK kinases which in turn sharply upregulates cyclin D1, leading to dysregulation of the G1/S checkpoint that plays a crucial role in breast cancer, being overexpressed in 40% of most cases [52]. Cyclin D1 can also be activated by PRL/PRLR signaling through MAPK. This clearly shows that signaling cascades activated by PRLR, EGFR and HER2 overlap and share common kinases that can induce multiple downstream effectors/target genes.

Multiple evidence has shown crosstalk between PRLR and EGFR signaling pathways in breast cancer. PRL and EGF via their cognate receptors synergistically induce MEK/ERK1/2 and PI3K/AKT pathways which promote proliferation, survival, and invasion of breast cancer cells [67,68]. We have demonstrated that PRL/PRLR induces HER2 phosphorylation at Tyr residues 1221 and 1222 through JAK2, activation of downstream PI3K/AKT pathway in both MCF-7 and T47D cells [31]. This crosstalk between PRLR and HER2 signaling further facilitates the phosphorylation of ERα, its recruitment to the PRLR promoter and upregulation of PRLR transcription. Interestingly, we also found that EGF/EGFR in MCF-7 and T47D cells can induce PRLR transcription via downstream MAPK and PI3K signaling pathways leading to phosphorylation of ERα at Ser167 and Ser118 and phosphorylation of STAT5b [69]. EGFR can bind to and activate STAT5b indirectly with participation of c-SRC which phosphorylates EGFR at Tyr 845 leading to recruitment of phospho-STAT5b along with pERα to the hPIII promoter and PRLR expression [69]. STAT5 can be also activated directly by EGFR through phosphorylation of STAT5 in an EGF-dependent manner at specific sites. Activated STAT5 translocate into the nucleus to drive the expression of specific target genes involved in proliferation, differentiation, and survival. Further, increased EGFR and HER2 activity was known to promote constant activation of STAT3, which leads to tumor progression and survival [53,70]. PRL promotes cell motility in several breast cancer cell lines mediated through EGF [71]. A study from Huang et al. indicates that PRL/PRLR can also induce ERK dependent EGFR and HER2 phosphorylation at different sites detected by PTP101 antibody that recognizes threonine phosphorylation at consensus motifs for ERK-induced phosphorylation. This was evident by PD98059, a MEK inhibitor that prevented PRL induced PTP101 threonine phosphorylation of EGFR and HER indicating PRL induced phosphorylation was dependent on the ERK pathway [67]. 

**Figure 3:**
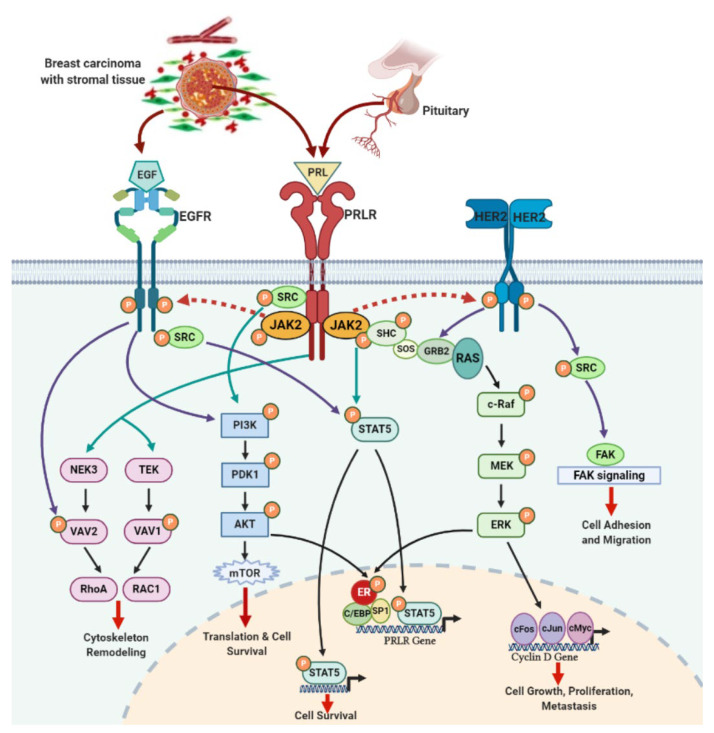
PRLR and EGFR/HER2 signaling crosstalk in breast cancer: PRL secreted by pituitary and breast tumor cells activates the PRLR downstream signaling pathways such as JAK/STAT, RAS/MEK/ERK, PI3K/AKT, NEK3-VAV2 and TEC-VAV1 responsible for proliferation, survival, and metastasis of breast tumor. EGF released by stromal microenvironment surrounding the breast tumor activates EGFR signaling kinases that overlap with PRLR signaling. RAS/MEK/ERK and PI3K/AKT signaling can activated unliganded ER which forms complex with Sp1/C/EBPβ and recruited along with STAT5 at the PRLR promoter. HER2 overexpression in breast cancer cells can activate RAS/MEK/ERK and c-SRC/FAK signaling pathways. PRL/PRLR signal activates both EGFR and HER2 through phosphorylation via JAK2 which leads to activation of these signaling pathways. EGFR can activate STAT5 signaling directly or indirectly via s-SRC. Such a crosstalk between these two receptors can further intensify the tumor progression. This image was created using Biorender software.

Interestingly, this study showed that PRL synergized with EGF to activate SHC/ERK/c-FOS cascade, and this was prevented by ERK inhibitor suggesting that PRL facilitated the markedly enhanced EGF signaling. Taken together, we have dissected existing evidence on the PRLR and EGFR/HER2 signaling crosstalk and how their downstream signaling kinases interact with each other to activate several target genes like unliganded ER, RhoA, RAC1 and other pro-oncogenic factors like FOS, JUN, MYC. These interconnected downstream signaling pathways activated by EGFR/HER2 and PRLR cooperation may enhance proliferation, survival, migration, and invasiveness of breast cancer cells (Figure 3).

## 5. Conclusions and Future Perspectives

In this review, we have provided evidence to support the significant role for PRLR and EGFR and their crosstalk signaling in breast tumor development, growth, and survival. PRL via PRLR can activate HER2 downstream signaling pathways through JAK2 signal and this crosstalk with help of other downstream cascades can further enhance the activation of ER independent of estrogen. In addition to this, PRL can synergistically augment EGF/EGFR signaling in breast cancer cells by activating ERK and requires threonine phosphorylation of EGFR. Both PRLR and EGFR can interact and activate with STAT5 directly, which in turn drives expression of downstream targets c-MYC, cyclin D and others leading to tumor proliferation and survival. PRLR and EGFR/HER2 crosstalk which greatly increases the activation of the RAS/ERK and PI3K/AKT pathways are associated with a poor prognosis and therapeutic resistance in breast tumor patients. A significant number of breast cancer patients develop resistance to endocrine therapies often resulting in tumor relapse. Resistance to endocrine therapies is associated with enhanced MAPK and PI3K signaling pathways, overexpression of HER2, and activation of other oncogenic growth factors. Even with successful treatment of trastuzumab a significant portion of patients develop resistance to this drug and exhibit cancer relapse within 1 year of treatment. Hyperactivation of HER2-downstream PI3K/Akt pathway was often observed in trastuzumab-resistant breast cancer patients [72]. In addition, mutations in HER2 gene are also a well-known intrinsic and acquired mechanisms of resistance. These can be HER2 activating mutations, and other mutations can prevent binding of trastuzumab. Multiple HER2-targeted therapies have been established over the past few years, including the tyrosine kinase inhibitors such as lapatinib, neratinib, tucatinib, and pyrotinib. These drugs which target multiple receptors (EGFR, HER2 and HER4) altogether were studied in the early and advance stages of breast cancer and have shown some promising results [73]. In the case of PRLR, there have been only few successful attempts in developing a potential therapeutic small molecule inhibitors or monoclonal antibody to block PRLR signaling induced cell proliferation in breast cancer cell lines. Recently, two small molecule inhibitors (SMI-1 & SMI-6) were identified using high throughput screening of small molecules that are capable of binding to the extracellular domain of PRLR and abrogated PRL-induced cell invasion and malignant lymphocyte proliferation. Further, SMI-6 dramatically reduced tumor growth in PRL expressing breast cancer xenografts [74]. A phase-I clinical study of LFA102, a humanized monoclonal antibody (mAb) that binds PRLR did not show favorable outcome although it was found to be very effective in pre-clinical trial [75]. Therefore, simultaneous treatments targeting the PRLR and HER2 pathway may provide better outcome in effectively inhibiting breast tumor growth and ameliorate endocrine resistance/relapse period. A study using G129R (PRLR antagonist) and trastuzumab (monoclonal antibody targeting HER2) as combination therapy on inhibition of HER2+ breast cancer cells and nude mice xenograft model showed inhibition of cell proliferation [76]. Also, combining PI3K/AKT/mTOR pathway inhibitors with endocrine therapy has been shown to potentially reverse resistance to trastuzumab in HER2+ patients and metastatic breast cancer in early clinical trials [77]. A rational combination of therapeutic agents based on the disease profile would be more beneficial to breast cancer patients.
